# Challenges in breast cancer management and prevention

**DOI:** 10.1186/1878-5085-5-S1-A41

**Published:** 2014-02-11

**Authors:** Manuel Debald, Walther Kuhn, Olga Golubnitschaja

**Affiliations:** 1 Department of Obstetrics and Gynaecology, Centre for Integrated Oncology, University of Bonn, Germany; 2 Breast Cancer Research Centre, University of Bonn, Germany; 3 Department of Radiology, Rheinische Friedrich-Wilhelms-University of Bonn, Sigmund-Freud-Str. 25, Germany

## 

Breast cancer is the most frequently diagnosed cancer entity and the leading cause of cancer death in females worldwide, accounting for 23% of the total new cancer cases and 14% of the total cancer deaths in 2008 [1]. Recently, prophylactic mastectomy in patients with family history for breast cancer or patients with BRCA1 or BRCA2 mutations became a widely discussed topic due to the wide media presence of celebrities undergoing this procedure. Prophylactic mastectomy in patients with BRCA 1/2 mutations reduces the risk for breast cancer by approximately 90%. Nevertheless, young patients undergoing this risk reducing surgery carry many fears and psychological stress by feeling less female and are faced by a life-changing decision. In addition, several studies demonstrated that not all patients with a breast cancer history do really benefit from prophylactic surgery of the contralateral breast that trigger controversial discussions regarding optimized breast cancer management [2].

Economic burden of breast cancer management are permanently increasing negatively impacting the healthcare budgets. In the actual context of extensive debates concerning increasing costs of medical services and limited resources to cover healthcare costs, new strategies needs to be applied to more effective breast cancer management. To give an example, in the USA the costs for prescriptions against breast cancer are the second largest category of all pharmaceutical sales with enormously increasing rates. The therapy costs for each patient with metastatic breast cancer have been reported to be $128.556 over a mean follow-up time of 18 month. But also the trend towards the detection of earlier breast cancer stages is under discussion regarding treatment savings. The savings of costs for treatment and palliative care in advanced breast cancer may be counterbalanced by the high costs of more aggressive initial treatments and longer follow-up.

Currently applied screening strategies lack sufficient benefits and, therefore, also become disputed. The diagnosis of breast cancer in early stages has been shown to improve the prognosis and consecutively reduce mortality of breast cancer patients. The recommended screening by clinical breast examination and mammography is able to detect breast cancer in early stages and has been shown to reduce mortality. Nevertheless, this screening procedures cause high percentages of false-negative and false-positive results that lead to both underdiagnosis and overdiagnosis respectively [3]. Furthermore, established mammography screening programs are restricted to certain age-groups (50-69 years) and specialized screening units and not feasible in most economically developing countries.

## Recommendations

Based on the enormous amount of health care costs for breast cancer screening, therapy costs as well as the economic burden of this disease, predictive medicine could be an innovative approach towards a new era in breast cancer prevention. Breast cancer risk assessment aims at detection of pre-malignant stages that will revolutionise breast cancer management in patients without family history as well as in women with hereditary disease. Innovative approaches proposed by the PPPM paradigm promote economically more attractive scenario for the investment in healthcare sector, however, should get carefully analysed through well designed pilot projects [4,5].
